# Risk Factors and Outcomes of Non-*albicans Candida* Bloodstream Infection in Patients with Candidemia at Siriraj Hospital—Thailand’s Largest National Tertiary Referral Hospital

**DOI:** 10.3390/jof7040269

**Published:** 2021-04-01

**Authors:** Chaiyapong Ngamchokwathana, Piriyaporn Chongtrakool, Amiroh Waesamaae, Methee Chayakulkeeree

**Affiliations:** 1 Department of Medicine, Faculty of Medicine Siriraj Hospital, Mahidol University, Bangkok 10700, Thailand; octofive@gmail.com; 2 Department of Microbiology, Faculty of Medicine Siriraj Hospital, Mahidol University, Bangkok 10700, Thailand; piriyaporn.cho@mahidol.ac.th; 3 Research Department, Faculty of Medicine Siriraj Hospital, Mahidol University, Bangkok 10700, Thailand; amiroh.wae@gmail.com; 4 Department of Medicine, Division of Infectious Diseases and Tropical Medicine, Faculty of Medicine Siriraj Hospital, Mahidol University, Bangkok 10700, Thailand

**Keywords:** risk factors, outcomes, non-*albicans Candida*, candidiasis, candidemia, *Candida*, Thailand

## Abstract

This study aimed to investigate the risk factors for and the outcomes of patients with candidemia caused by non-*albicans Candida*. Candidemia patients treated at Siriraj Hospital (Bangkok, Thailand) during January 2016 to December 2017 were enrolled. A total of 156 patients (mean age: 65 years, 56.4% male) were included. The most prevalent underlying conditions were diabetes (32.1%), chronic cardiac disease (28.2%), chronic kidney disease (26.9%), and hematologic malignancies (21.2%). *Candida* species isolated from patient blood were *C. tropicalis* (49.4%), *C. albicans* (28.8%), *C. glabrata* (16.7%), and *C. parapsilosis* (5.1%). Fluconazole resistance was significantly increased in *C. tropicalis* (37.8%). No independent risk factors were associated with patients with non-*albicans Candida* candidemia compared to those with *C. albicans* candidemia. There was no significant difference in mortality between patients with non-*albicans Candida* candidemia and patients with *C. albicans* candidemia (OR: 1.35, 95% CI: 0.64–2.85). When compared with *C. albicans* candidemia, multivariate analysis revealed chronic liver disease (OR: 11.39, 95% CI: 1.38–94.02), neutropenia (OR: 4.31, 95% CI: 1.34–13.87), and male gender (OR: 2.34, 95% CI: 1.04–5.29) to be independent risk factors for *C. tropicalis* candidemia. The observed high resistance of *C. tropicalis* to fluconazole indicates that fluconazole should not be used for empirical antifungal treatment in these patients.

## 1. Introduction

*Candida* spp. is the most common fungal pathogen that causes nosocomial infection [1,2]. Candidemia, which is a blood stream infection caused by *Candida*, is a serious public health problem that is associated with high mortality and high medical and economic costs [2]. A previous study reported the incidence of candidemia to be approximately 13.3 per 100,000 patients [3], and the mortality rate ranged from 36% to 59% [4,5]. The incidence of *Candida* infection increases following advanced medical interventions, such as hematopoietic stem cell transplantation and solid organ transplantation, as well as after invasive medical procedures, such as the use of vascular catheter or mechanical ventilator [4]. Factors reported to be associated with mortality in candidemia included neutropenia, septic shock, intensive care unit (ICU) admission, inappropriate antifungal therapy within 72 h, and renal failure [5]. 

Previous epidemiological studies demonstrated the same trend toward increasing numbers of patients with candidemia in both neutropenic and non-neutropenic individuals. The incidence of non-*albicans Candida* has risen dramatically in some countries, especially in Asia, and there has been a shift from *Candida albicans* to non-*albicans Candida* species as the more prevalent causative pathogen in candidemia patients [6,7,8,9,10,11]. *Candida tropicalis* is the most common non-*albicans Candida* species in tropical countries, including Thailand [12]. Although fluconazole resistance is commonly found in *Candida glabrata* [13], *C. tropicalis* isolates exhibit decreased susceptibility to azole agents [13,14]. The mortality rate in candidemia may vary according to *Candida* species [15]. However, the mortality rate in candidemia caused by non-*albicans Candida* is relatively higher than the mortality rate in candidemia caused by *C. albicans* [16]. Early appropriate antifungal treatment significantly improves outcomes, and it reduces the high hospital costs normally associated with candidemia [17]. Delay in the initiation of fluconazole therapy in hospitalized patients with candidemia significantly adversely influenced mortality [18].

Despite the existing data from previous studies, the risk factors and clinical outcomes of patients with bloodstream infection from non-*albicans Candida* are still unclear. Accordingly, the aim of this study was to identify the independent risk factors for candidemia caused by non-*albicans Candida*, particularly *C. tropicalis*. The secondary objective was to investigate antifungal susceptibility and mortality among non-*albicans Candida* candidemia patients.

## 2. Materials and Methods

### 2.1. Study Design 

This retrospective cohort study included candidemia patients diagnosed and treated at the Faculty of Medicine Siriraj Hospital, Mahidol University, Bangkok, Thailand during January 2016 to December 2017. Siriraj Hospital is a 2300-bed national super-tertiary referral center. This study was registered in the Thai Clinical Trials Registry (ID: TCTR20190501003).

### 2.2. Study Population

All candidemia patients aged 18 years or older who had a positive blood culture for *Candida* spp. were enrolled. Patients whose blood culture grew more than 1 species of *Candida*, who had recurrent candidemia, and/or who had an incomplete medical record were excluded. 

Collected patient demographic and clinical data included age; gender; duration of hospital stay; and risk factors for candidemia, including central venous catheter, mechanical ventilation, parenteral nutrition, hemodialysis, peritoneal dialysis, recent abdominal surgery, ICU admission, urinary catheter, prosthesis, broad spectrum antibiotics, corticosteroid equivalent to prednisolone at least 20 mg/day for at least 2 weeks, absolute neutrophil count < 500 cells/mm^3^, yeast in non-sterile body samples, prior antifungal exposure, and comorbidities. Candidemia was categorized into primary fungemia, acute disseminated candidiasis, catheter-related blood stream infection, intra-abdominal infection, hepatosplenic abscess, bone and joint infection, central nervous system infection, endocarditis, and urinary tract infection. All *Candida* isolated from blood cultures were identified to the species level. The species identification of *Candida* was performed based on biochemical characteristics using chromogenic medium (Brilliance™ Candida agar) and RapID™ Yeast Plus Panel (Thermo Fisher Scientific, Kent, UK). Antifungal susceptibility was tested by the microbroth dilution method using Thermo Scientific™ Sensititre™ YeastOne™ YO9 AST Plate (Thermo Fisher Scientific, Waltham, MA, USA). Minimal inhibitory concentration (MIC) breakpoints were interpreted according to the Clinical Laboratory Standards Institute (CLSI). Antifungal susceptibility test results and clinical outcomes were analyzed. 

### 2.3. Statistical Analysis 

Continuous data are presented as mean ± standard deviation (SD) for normally distributed data, and as median and interquartile range (IQR) for non-normally distributed data. Categorical data are presented as frequency and percentage. The Chi-square test or Fisher’s exact test was used for the comparison of categorical variables between two groups. The independent *t*-test was used to compare normally distributed continuous data, and the Mann–Whitney U test was used to compare non-normally distributed continuous data. Multivariate analysis via multiple logistic regression model (backward stepwise) included all variables with a *p*-value less than 0.2 from univariate analysis. All statistical analyses were performed using SPSS Statistics (v18.0) software (SPSS, Inc., Chicago, IL, USA), and a 2-sided *p*-value less than 0.05 was considered to be statistically significant.

## 3. Results 

### 3.1. Patient Data

A total of 156 patients were enrolled. The mean age was 64 years, and 56.4% of patients were male. The most prevalent underlying conditions were diabetes mellitus (32.1%), chronic cardiac disease (28.2%), chronic kidney disease (26.9%), and hematologic malignancies (21.2%) (Table 1).

### 3.2. Candida Species Distribution and Antifungal Susceptibility

**Figure 1:**
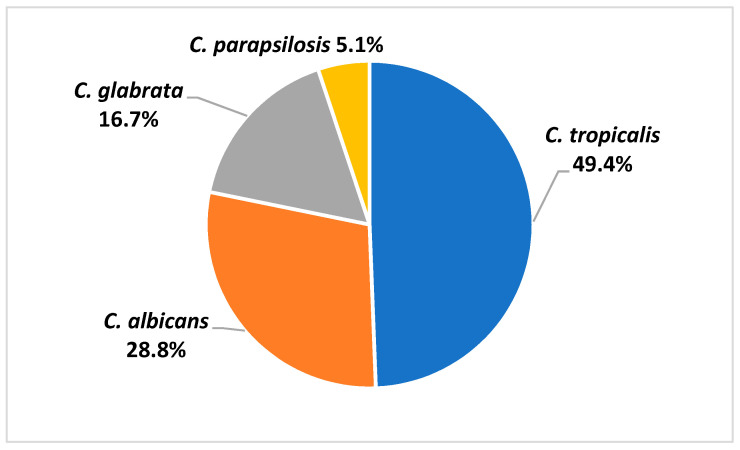
Species distribution of *Candida* isolated from blood samples of patients with candidemia.

**Figure 2:**
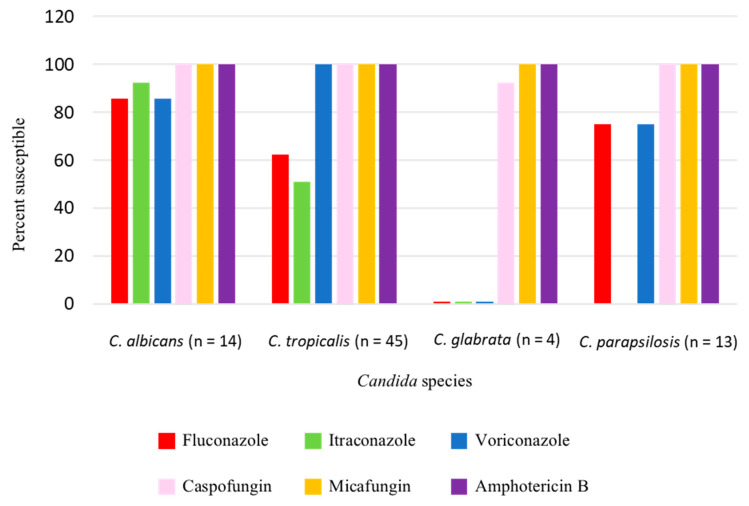
Antifungal susceptibility of *Candida* isolates.

The *Candida* species isolated from patient blood were *C. tropicalis* (49.4%), *C. albicans* (28.8%), *C. glabrata* (16.7%), and *C. parapsilosis* (5.1%) (Figure 1). Antifungal susceptibility results were available in 76 *Candida* isolates. Table 2 demonstrated the minimal inhibitory concentration (MIC) range, MIC50, and MIC90 of antifungal agents for each *Candida* species in this study. Fluconazole resistance was higher in *C. tropicalis* (37.8%) than in *C. albicans* (14.3%) (Figure 2).

### 3.3. Factors Significantly and Independently Associated with Non-albicans Candida Candidemia

Patient characteristics and other factors significantly associated with non-*albicans Candida* and *C. albicans* candidemia are shown in Table 1 and Table 3. Chronic liver disease and neutropenia were the only two factors found to be significantly associated with non-*albicans Candida* candidemia in univariate analysis.

Subgroup analysis of candidemia caused by *C. tropicalis* or *C. albicans* to identify the factors associated with those two infections is shown in Table 4 and Table 5. Multivariate analysis revealed male gender, chronic liver disease, and neutropenia to be factors independently associated with *C. tropicalis* infection (Table 6). The mortality rate was not significantly different among patients with candidemia caused by *C. albicans*, non-*albicans Candida*, or *C. tropicalis*.

## 4. Discussion

From January 2016 to December 2017, we analyzed data from 156 candidemia patients admitted to Siriraj Hospital, the largest university hospital in Thailand. Most patients had candidemia that was caused by non-*albicans Candida* (71.2%), which is consistent with a previous study in Thailand, and which reveals an increasing incidence of non-*albicans* candidemia [4]. *C. tropicalis* was found to be the most common *Candida* species in patients with candidemia, and this is similar to the results of previous studies conducted at the same hospital [5,19]. It was proposed that the pathogenicity of *C. tropicalis* may be associated with a warmer climate [12], which may explain why it was found to be the most common *Candida* species in our study.

The present study identified neutropenia and chronic liver disease as potential risk factors for non-*albicans Candida* candidemia. A previous study also reported non-*albicans Candida* candidemia to be associated with neutropenic patients [6]. However, no previous study has reported chronic liver disease as a risk factor of non-*albicans Candida* candidemia.

Risk factors associated with *C. tropicalis* and *C. albicans* candidemia were also analyzed in this study. The results of that analysis revealed neutropenia, chronic liver disease, and male gender to be independent risk factors for *C. tropicalis* candidemia. A previous study reported the independent risk factors for *C. tropicalis* candidemia to be abdomen portal entry, hematologic malignancies, and older age [20]. Our study confirmed that *C. tropicalis* seems to originate from the intra-abdominal origin, and it tends to infect neutropenic patients.

*Candida tropicalis* has become increasingly resistant to fluconazole. Two previous studies that were conducted at our center in 2009 and 2013 found rates of *C. tropicalis* susceptibility to fluconazole of 100% and 70.3%, respectively [5,19]. However, in this study, we found a rate of *C. tropicalis* susceptibility to fluconazole of only 62%. Therefore, empirical antifungal treatment with echinocandins or amphotericin B product in candidemia patients with risk factors for *C. tropicalis* infection is strongly recommended. In our study, most of the patients infected with fluconazole-resistant *C. tropicalis* did not have a history of azole exposure and they may be acquired from the environment [21]. Azoles have been used in agriculture in Thailand and the link between fluconazole-resistant *Candida* and environmental azole exposure needs to be investigated.

The mortality rate among candidemia patients in this study was approximately 70%, which is higher than in other studies, but the mortality rate was not found to be significantly different between *C. albicans* and non-*albicans Candida* candidemia. This extremely high mortality rate in candidemia patients highlights the need to improve candidemia management guidelines in Thailand.

The limitations of this study include its single-center retrospective cohort design, the fact that the epidemiology of *Candida* species can differ among hospitals, and that antifungal susceptibility testing was not routinely performed in every included study patient.

## 5. Conclusions

In candidemia patients, chronic liver disease, neutropenia, and male gender were found to be independently associated with *C. tropicalis* infection. Due to the observed high resistance of *C. tropicalis* to fluconazole, fluconazole should not be used for empirical antifungal treatment in these patients.

## Figures and Tables

**Table 1:** Characteristics of patients with candidemia caused by non-*albicans Candida* and *Candida albicans.*

Characteristics	Non-*albicans Candida* (*n* = 111)	*C. albicans* (*n* = 45)	Odds Ratio (95% CI)	*p*
Male gender, *n* (%)	58 (52.2%)	30 (66.7%)	0.54 (0.27–1.13)	0.1
Age (years), mean ± SD	63. ± 18	67 ± 18	-	0.302
LOS (days), median (IQR)	8 (2–26)	10 (3–19)	-	0.977
Diagnosis, *n* (%)				
Primary fungemia	60 (54.1%)	19 (42.2%)	1.61 (0.80–3.24)	0.181
CRBSI	41 (36.9%)	19 (42.2%)	0.80 (0.40–1.62)	0.539
Intra-abdominal infection	7 (6.3%)	5 (11.1%)	0.54 (0.16–1.80)	0.329
Hepatosplenic abscess	1 (0.9%)	0 (0.0%)	-	1
Endocarditis	1 (0.9%)	0 (0.0%)	-	1
Urinary tract infection	1 (0.9%)	2 (4.4%)	0.20 (0.01–2.21)	0.2
Comorbidities, *n* (%)				
Chronic cardiac disease	30 (27.0%)	14 (31.1%)	0.82 (0.38–1.75)	0.608
Chronic lung disease	15 (13.5%)	10 (22.2%)	0.55 (0.22–1.33)	0.179
Chronic kidney disease	27 (24.3%)	15 (33.3%)	0.64 (0.30–1.36)	0.25
Chronic liver disease	15 (13.5%)	1 (2.2%)	6.89 (0.88–52.63)	0.035
Diabetes mellitus	37 (33.3%)	13 (28.9%)	1.23 (0.58–2.62)	0.59
HIV disease	2 (1.8%)	1 (2.2%)	0.80 (0.07–9.17)	1
Autoimmune disease	10 (9.0%)	5 (11.1%)	0.79 (0.25–2.46)	0.687
Hematologic malignancies	28 (25.2%)	5 (11.1%)	2.69 (0.97–7.51)	0.051
HSCT	2 (1.8%)	0 (0.0%)	-	1
Solid organ transplantation	0 (0.0%)	1 (2.2%)	-	0.288
Mortality	81 (73.0%)	30 (66.7%)	1.35 (0.64–2.85)	0.431

A *p*-value < 0.05 indicates statistical significance; Abbreviations: SD, standard deviation; LOS, length of stay; IQR, interquartile range; CRBSI, catheter-related blood stream infection; HIV, human immunodeficiency virus; HSCT, hematopoietic stem cell transplantation.

**Table 2:** Minimal inhibitory concentration (MIC) range, MIC50, and MIC90 of antifungal agents for each *Candida* species.

Species	Antifungal Agent	MIC Range (mg/L)	MIC50 (mg/L)	MIC90 (mg/L)
*Candida albicans*	Amphotericin B	0.25–1	0.5	1
	Fluconazole	0.25–256	0.25	1
	Itraconazole	0.025–0.25	0.06	0.12
	Voriconazole	≤0.008–2	≤0.008	0.03
	Posaconazole	≤0.008–0.25	0.03	0.06
	5-fluorocytosine	≤0.06–0.12	≤0.06	0.12
	Caspofungin	0.03–0.06	0.06	0.06
	Micafungin	≤0.008–0.03	≤0.008	0.02
*Candida tropicalis*	Amphotericin B	0.5–2	1	1
	Fluconazole	≤0.12–256	2	256
	Itraconazole	0.06–1	0.25	1
	Voriconazole	0.06–4	0.12	0.5
	Posaconazole	0.06–1	0.25	1
	5-fluorocytosine	≤0.06–0.25	≤0.06	0.06
	Caspofungin	0.03–0.25	0.03	0.12
	Micafungin	0.03–0.5	0.03	0.03
*Candida parapsilosis*	Amphotericin B	0.5–1	1	1
	Fluconazole	0.25–8	1	8
	Itraconazole	0.06–0.12	0.06	0.12
	Voriconazole	≤0.008–0.25	0.02	0.25
	Posaconazole	0.03–0.06	0.03	0.06
	5-fluorocytosine	≤0.06–0.12	0.06	0.12
	Caspofungin	0.25–0.5	0.25	0.5
	Micafungin	0.5–1	0.5	1
*Candida glabrata*	Amphotericin B	0.5–2	1	1
	Fluconazole	2–32	8	32
	Itraconazole	0.25–1	0.5	1
	Voriconazole	0.12–1	0.25	1
	Posaconazole	0.25–2	1	2
	5-fluorocytosine	≤0.06	≤0.06	≤0.06
	Caspofungin	0.03–0.25	0.06	0.12
	Micafungin	≤0.008–0.03	0.03	0.03

**Table 3:** Univariate analysis for factors significantly associated with non-*albicans Candida* and *Candida albicans* infection in patients with candidemia.

Risk Factors	Non-*albicans Candida* (*n* = 111), *n* (%)	*C. albicans* (*n* = 45), *n* (%)	Odds Ratio (95% CI)	*p*
Central venous catheterization	73 (65.8%)	33 (73.3%)	0.70 (0.32–1.51)	0.359
Mechanical ventilator	81 (73.0%)	36 (80.0%)	0.68 (0.29–1.56)	0.358
Parenteral nutrition	36 (32.4%)	12 (26.7%)	1.32 (0.61–2.86)	0.480
Hemodialysis	46 (41.4%)	18 (40.0%)	1.06 (0.52–2.15)	0.868
Peritoneal dialysis	3 (2.7%)	0 (0.0%)	-	0.557
Recent abdominal surgery	19 (17.1%)	7 (15.6%)	1.12 (0.44–2.88)	0.813
ICU admission	65 (58.6%)	25 (55.6%)	1.13 (0.56–2.27)	0.731
Urinary catheterization	93 (83.6%)	40 (88.9%)	0.65 (0.22–1.86)	0.415
Presence of prosthesis	12 (10.8%)	9 (20.0%)	0.48 (0.19–1.25)	0.128
Carbapenem use	59 (53.2%)	21 (46.7%)	1.30 (0.65–2.30)	0.463
Cephalosporin use	18 (16.2%)	8 (17.8%)	0.90 (0.36–2.24)	0.813
Fluoroquinolone use	13 (11.7%)	9 (20.0%)	0.53 (0.21–1.35)	0.178
Corticosteroid use	12 (10.8%)	7 (15.6%)	0.66 (0.24–1.80)	0.412
Neutropenia	25 (22.5%)	4 (8.9%)	2.97 (0.97–9.09)	0.047
Presence of yeast in urine	56 (50.5%)	23 (51.1%)	0.91 (0.42–1.96)	0.816
Presence of yeast in sputum	49 (44.1%)	24 (53.3%)	0.68 (0.31–1.51)	0.342
Presence of yeast in feces	6 (5.4%)	1 (2.2%)	2.07 (0.22–19.23)	0.517
Antifungal exposure within 1 month	4 (3.6%)	2 (4.4%)	0.80 (0.14–4.55)	1.000
Azoles exposure within 1 month	4 (3.6%)	1 (2.2%)	1.64 (0.18–15.15)	1.000

A *p*-value < 0.05 indicates statistical significance; Abbreviation: CI, confidence interval; ICU, intensive care unit.

**Table 4:** Characteristics of patients with candidemia caused by *Candida tropicalis* or *Candida albicans.*

Factors	*C. tropicalis* (*n* = 77)	*C. albicans* (*n* = 45)	Odds Ratio (95% CI)	*p*
Male gender, *n* (%)	58 (75.3%)	30 (66.7%)	1.95 (0.91–4.18)	0.085
Age (years), mean ± SD	62 ± 18	67 ± 18	-	0.302
LOS (days), median (IQR)	7 (2–18)	10 (3–19)	-	0.977
Diagnosis, *n* (%)				
Primary fungemia	42 (54.5%)	19 (42.2%)	1.64 (0.78–3.45)	0.181
CRBSI	30 (39.0%)	19 (42.2%)	0.87 (0.41–1.85)	0.539
Intra-abdominal infection	2 (2.6%)	5 (11.1%)	0.21 (0.04–1.15)	0.329
Hepatosplenic abscess	1 (1.3%)	0 (0.0%)	-	1
Endocarditis	1 (1.3%)	0 (0.0%)	-	1
Urinary tract infection	1 (1.3%)	2 (4.4%)	0.28 (0.03–3.21)	0.2
Comorbidities, *n* (%)				
Chronic cardiac disease	20 (26.0%)	14 (31.1%)	0.78 (0.35–1.75)	0.541
Chronic lung disease	10 (13.0%)	10 (22.2%)	0.52 (0.20–1.37)	0.184
Chronic kidney disease	17 (22.1%)	15 (33.3%)	0.57 (0.25–1.29)	0.173
Chronic liver disease	13 (16.9%)	1 (2.2%)	8.94 (1.13–70.82)	0.014
Diabetes mellitus	23 (29.9%)	13 (28.9%)	1.05 (0.48–2.35)	0.909
HIV disease	2 (2.6%)	1 (2.2%)	1.17 (0.10–13.32)	1
Autoimmune disease	8 (10.4%)	5 (11.1%)	0.93 (0.28–3.03)	1
Hematologic malignancies	26 (33.8%)	5 (11.1%)	4.08 (1.44–11.57)	0.006
HSCT	2 (2.6%)	0 (0.0%)	-	1
Solid organ transplantation	0 (0.0%)	1 (2.2%)	-	0.288
Mortality	59 (76.7%)	30 (66.7%)	1.64 (0.73–3.70)	0.232

A *p*-value < 0.05 indicates statistical significance; Abbreviations: CI, confidence interval; SD, standard deviation; LOS, length of stay; IQR, interquartile range; CRBSI, catheter-related blood stream infection; HIV, human immunodeficiency virus; HSCT, hematopoietic stem cell transplantation.

**Table 5:** Univariate analysis for factors significantly associated with *Candida tropicalis* and *Candida albicans* infection in patients with candidemia.

Risk Factors	*C. tropicalis* (*n* = 77), *n* (%)	*C. albicans* (*n* = 45), *n* (%)	Odds Ratio (95% CI)	*p*
Central venous catheterization	49 (63.6%)	33 (73.3%)	0.64 (0.28–1.43)	0.271
Mechanical ventilator	53 (68.8%)	36 (80.0%)	0.55 (0.23–1.33)	0.180
Parenteral nutrition	22 (28.6%)	12 (26.7%)	1.10 (0.48–2.51)	0.821
Hemodialysis	34 (44.2%)	18 (40.0%)	1.19 (0.56–2.50)	0.654
Peritoneal dialysis	-	-	-	-
Recent abdominal surgery	11 (14.3%)	7 (15.6%)	0.91 (0.32–2.53)	0.849
ICU admission	45 (58.4%)	25 (55.6%)	1.13 (0.54–2.36)	0.756
Urinary catheterization	63 (81.8%)	40 (88.9%)	0.56 (0.19–1.68)	0.299
Presence of prosthesis	7 (9.1%)	9 (20.0%)	0.40 (0.14–1.16)	0.085
Carbapenem use	41 (53.2%)	21 (46.7%)	1.30 (0.62–2.72)	0.483
Cephalosporin use	9 (11.7%)	8 (17.8%)	0.61 (0.22–1.72)	0.349
Fluoroquinolone use	8 (10.4%)	9 (20.0%)	0.46 (0.17–1.30)	0.139
Corticosteroid use	9 (11.7%)	7 (15.6%)	0.72 (0.25–2.08)	0.541
Neutropenia	23 (29.9%)	4 (8.9%)	4.37 (1.40–13.61)	0.007
Presence of yeast in urine	40 (51.9%)	23 (51.1%)	0.93 (0.41–2.10)	0.864
Presence of yeast in sputum	35 (45.4%)	24 (53.3%)	0.79 (0.34–1.85)	0.587
Presence of yeast in feces	6 (7.8%)	1 (2.2%)	3.16 (0.33–30.00)	0.400
Antifungal exposure within 1 month	4 (5.1%)	2 (4.4%)	1.18 (0.21–6.70)	1.000
Azoles exposure within 1 month	3 (3.9%)	1 (2.2%)	1.78 (0.18–17.68)	1.000

A *p*-value < 0.05 indicates statistical significance; Abbreviation: CI, confidence interval; ICU, intensive care unit.

**Table 6:** Multivariate analysis for factors independently associated with *Candida tropicalis* and *Candida albicans* infection in patients with candidemia.

Risk Factors	*C. tropicalis* (*n* = 77), *n* (%)	*C. albicans* (*n* = 45), *n* (%)	Adjusted OR (95% CI)	*p*
Male gender	58 (75.3%)	30 (66.7%)	2.34 (1.04–5.29)	0.04
Chronic liver disease	13 (16.9%)	1 (2.2%)	11.39 (1.38–94.02)	0.024
Neutropenia	23 (29.9%)	4 (8.9%)	4.31 (1.34–13.87)	0.014
Intra-abdominal infection	2 (2.6%)	5 (11.1%)	0.36 (0.07–2.05)	0.252
Received fluoroquinolones at least 7 days	8 (10.4%)	9 (20.0%)	0.53 (0.17–1.63)	0.266
Presence of prosthesis	7 (9.1%)	9 (20.0%)	0.61 (0.19–1.94)	0.404
Chronic kidney disease	17 (22.1%)	15 (33.3%)	0.76 (0.304–1.92)	0.566
Hematologic malignancies	26 (33.8%)	5 (11.1%)	1.27 (0.25–6.47)	0.774
Chronic lung disease	10 (13.0%)	10 (22.2%)	0.92 (0.27–3.10)	0.893
Mechanical ventilator	53 (68.8%)	36 (80.0%)	1.03 (0.37–2.85)	0.958
Primary fungemia	42 (54.5%)	19 (42.2%)	1.02 (0.42–2.50)	0.958

A *p*-value < 0.05 indicates statistical significance; Abbreviation: OR, odds ratio; CI, confidence interval.

## Data Availability

No new data were created or analyzed in this study. Data sharing is not applicable to this article.
